# STAT3 Localizes in Mitochondria-Associated ER Membranes Instead of in Mitochondria

**DOI:** 10.3389/fcell.2020.00274

**Published:** 2020-04-22

**Authors:** Yixun Su, Xiaomin Huang, Zhangsen Huang, Taida Huang, Yunsheng Xu, Chenju Yi

**Affiliations:** ^1^The Seventh Affiliated Hospital of Sun Yat-sen University, Shenzhen, China; ^2^Department of Biochemistry, Yong Loo Lin School of Medicine, National University of Singapore, Singapore, Singapore

**Keywords:** STAT3, MAM, transcription factors, mitochondrial localization, ER

## Abstract

Signal transducer and activator of transcription 3 (STAT3) is a transcription factor (TF) that regulates a variety of biological processes, including a key role in mediating mitochondrial metabolism. It has been shown that STAT3 performs this function by translocating in minute amounts into mitochondria and interacting with mitochondrial proteins and genome. However, whether STAT3 localizes in mitochondria is still up for debate. To decipher the role of mitochondrial STAT3 requires a detailed understanding of its cellular localization. Using Percoll density gradient centrifugation, we surprisingly found that STAT3 is not located in the mitochondrial fraction, but instead, in the mitochondria-associated endoplasmic reticulum membrane (MAM) fraction. This was confirmed by sub-diffraction image analysis of labeled mitochondria in embryonic astrocytes. Also, we find that other TFs that have been previously found to localize in mitochondria are also found instead in the MAM fraction. Our results suggest that STAT3 and other transcriptional factors are, contrary to prior studies, consolidated specifically at MAMs, and further efforts to understand mitochondrial STAT3 function must take into consideration this localization, as the associated functional consequences offer a different interpretation to the questions of STAT3 trafficking and signaling in the mitochondria.

## Introduction

STAT3 is a TF encoded by the Stat3 gene in mouse. STAT3 has been found to be crucial to regulating a variety of biological processes such as embryonic development, immunogenic response, and carcinogenesis (Levy and Darnell, 2002). These processes occur through ligand-mediated activation of STAT3 such as through cytokines and growth factors. Structurally, STAT3 oligomerizes into homo- or hetero-dimers, translocating into the nucleus where it acts as a transcription activator in this form (Zhong et al., 1994; Levy and Darnell, 2002; Stark and Darnell, 2012).

Over the last decade, it has been reported that STAT3 can translocate into mitochondria, where it promotes mitochondrial respiration by interacting with various mitochondrial proteins, as well as the mitochondrial genome (Wegrzyn et al., 2009; Macias et al., 2014; Carbognin et al., 2016; Xu et al., 2016; Meier et al., 2017). Despite these findings, whether STAT3 localizes in mitochondria is still up for debate, and underlies further questions on how STAT3 can regulate mitochondrial metabolism.

In this report, we provide evidence that STAT3 does not exist in mitochondria but instead, localizes in MAM in the mouse brain, lung, and liver. MAM is a cellular structure formed by non-covalent protein interactions between the ER and mitochondria, which has broad implications in mitochondrial bioenergetics and reactive oxygen species production (Theurey and Rieusset, 2017; Rieusset, 2018). This suggests that STAT3 might regulate mitochondrial metabolism via MAM function. In addition, we show that other transcriptional factors that were previously reported to be in mitochondria only exist within the MAM fraction.

## Results

### STAT3 Does Not Localize to Pure Mitochondria

**FIGURE 1:**
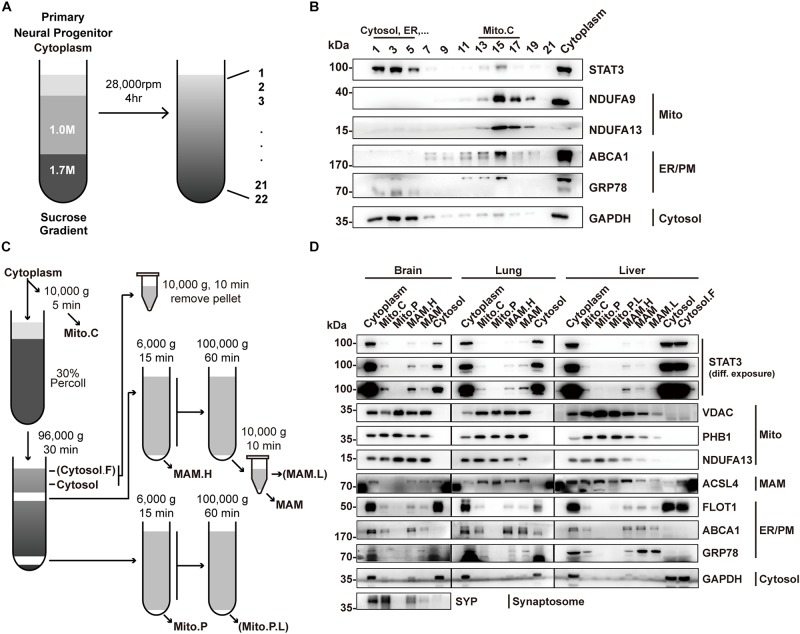
Percoll cell fractionation demonstrates that STAT3 does not exist in the mitochondria. **(A)** Experimental flow-chat for cell fractionation. **(B)** Western blots of cell fractionation. STAT3 was mainly found in Cytosol (fraction 1–5) and in crude mitochondria (fraction 13–17). **(C)** Experimental flow-chart of Percoll density centrifugation for MAM and pure mitochondria separation. Parentheses highlight fractions only obtained from liver. **(D)** Western blot of Percoll density centrifugation fractionation of brain, lung and liver. Pure mitochondria do not contain STAT3. The purity of mitochondria was confirmed by the absence of ER/PM marker. PM, plasma membrane; Mito.C, crude mitochondrial fraction; Mito.P, pure mitochondrial fraction; Mito.P.L., light pure mitochondria; MAM.H, heavy MAM; MAM.L, light MAM; Cytosol.F, cytosol fraction that contains fat; NDUFA9/13, NADH:ubiquinone oxidoreductase subunit A9/13; FLOT1, Flotillin-1; PHB1, Prohibitin 1.

To determine the localization of STAT3 in mitochondria, we first sought to isolate mitochondria from primary neural progenitor cells by sucrose gradient centrifugation (Figure 1A). STAT3 protein was detected in the mitochondria fraction, consistent with previous reports (Figure 1B). However, the presence of ER markers (GRP78 and ABCA1) and cytosol markers (GAPDH) suggests contamination by the MAM fraction (Figure 1B).

Percoll gradient centrifugation has been shown to be able to isolate pure mitochondria from those attached to the MAM (Vance, 1990). Thus, we attempted to use this method to isolate pure mitochondria fractions in different mouse tissues including the brain, lung, and liver (Figure 1C). Western blot results showed that the pure mitochondrial fraction contained no detectable STAT3 protein in all three tissues, suggesting that STAT3 does not exist in mitochondria. The absence of ER and cytosol markers confirmed the successful isolation of pure mitochondria, while the existence of mitochondrial outer membrane protein VDAC suggested the integrity of the pure mitochondria (Figure 1D).

Instead, STAT3 protein was found in the fractions containing MAM, which was confirmed by the immunoblot of mitochondria marker, ER marker as well as the MAM enriched protein ACSL4 (Figure 1D). ACSL4 showed different subcellular localization in different tissues. In brain it was also found in the cytosol fraction (which also contains membrane compartment such as ER and plasma membrane), while in lung and liver it also localized in the pure mitochondria fraction, similar to the fractionation result of previous publications (Sala-Vila et al., 2016; Radif et al., 2018).

Mitochondria-associated endoplasmic reticulum membrane isolated from brain could be contaminated by synaptosome. Immunoblot of synaptosome marker synaptophysin (SYP) showed that the MAM fraction might be contaminated by synaptosomes. That being said, the localization of STAT3 in the MAM fraction could still be confirmed in other tissues.

### Immunofluorescence Confirmed the Absence of STAT3 in Mitochondria

**FIGURE 2:**
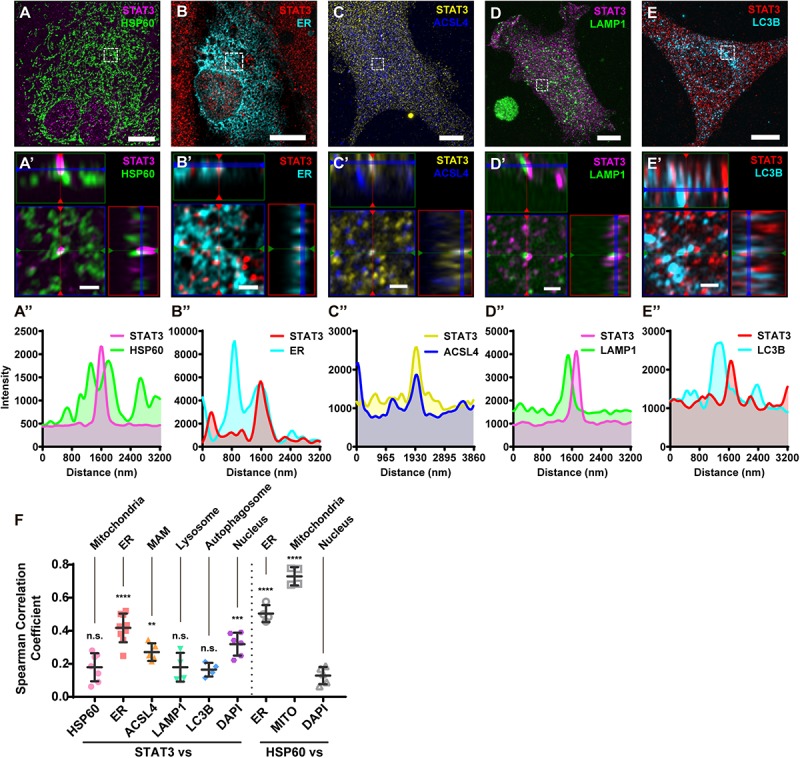
Immunofluorescence of primary astrocytes demonstrate that STAT3 does not colocalize with mitochondria. **(A)** Representative sub-diffraction maximum-intensity-projection image of immunofluorescence labeling of STAT3 and HSP60 in primary astrocytes (Scale bar: 10 um). **(A′)** The orthogonal views of the highlighted areas (Scale bar: 1 um). **(A′′)** Profiling of STAT3 and HSP60 signal intensity along green line in **(A′)**. **(B)** Representative image of STAT3 and ER-dsRed (ER) co-labeling (Scale bar: 10 um). **(B′)** The orthogonal views of the highlighted areas (Scale bar: 1 um). **(B′′)** Profiling of STAT3 and ER signal intensity along green line in **(B′)**. **(C)** Representative image of STAT3 and ACSL4 co-staining (Scale bar: 10 um). **(C′)** The orthogonal views of the highlighted areas (Scale bar: 1 um). **(C′′)** Profiling of STAT3 and ACSL4 signal intensity along green line in **(C′)**. **(D)** Representative image of STAT3 and LAMP1 co-staining (Scale bar: 10 um). **(D′)** The orthogonal views of the highlighted areas (Scale bar: 1 um). **(D′′)** Profiling of STAT3 and LAMP1 signal intensity along green line in **(D′)**. **(E)** Representative image of STAT3 and LC3B co-staining (Scale bar: 10 um). **(E′)** The orthogonal views of the highlighted areas (Scale bar: 1 um). **(E′′)** Profiling of STAT3 and LC3B signal intensity along green line in **(E′)**. **(F)** Spearman correlation coefficients calculated by Coloc2 (*N* = 2, *n* ≥ 2). Statistic test was conducted to evaluate the difference between each group and the negative control (HSP60 vs. DAPI) ****p < 0.0001, ***p < 0.001, **p < 0.005, n.s., not significant; MITO, Mitotracker DeepRed.

To further illustrate that STAT3 does not exist in mitochondria, we performed immunofluorescence studies on primary astrocytes. Using sub-diffraction image analysis (Zeiss Airyscan), we found that STAT3 did not colocalize with the mitochondrial marker HSP60 (Figure 2A). Small amounts of STAT3 were found to localize near mitochondria, which cannot be resolved using normal confocal microscopy methods (Figure 2A′,A′′). In contrast, STAT3 was found to colocalize with the ER (Figure 2B) as well as the MAM enrich protein ACSL4 (Figure 2C). MAM also tether to other membrane organelles such as lysosome and autophagosome (Hamasaki et al., 2013; Atakpa et al., 2018). Thus, we went on to examine if STAT3 also exists within lysosome and autophagosome. Results showed that STAT3 colocalized neither with lysosomal-associated membrane protein 1 (LAMP1), a lysosome marker, nor with microtubule associated protein 1 light chain 3 beta (LC3B), an autophagosome marker (Figures 2D,E). Quantification of colocalization using the Coloc 2 Image J plugin also showed that the correlation coefficient between STAT3 and ER, STAT3 and ACSL4 was higher than that between STAT3 and HSP60, LAMP1, and LC3B. The latter was similar to that of the negative control (DAPI and HSP60) (Figure 2F). Together, immunofluorescence results confirm the absence of STAT3 in mitochondria and its existence in MAM.

### Other Methods to Examine STAT3 Localization in Mitochondria Failed to Completely Remove MAM

In prior work done by other groups, several pure mitochondria isolation methods have been used to evaluate the localization of STAT3 in mitochondria, including trypsinization and sonication. We sought to examine whether these methods can reliably dissociate the MAM fractions from the pure mitochondria fractions, and analyze whether STAT3 localizes with ER/cytosol markers in these fractions.

**FIGURE 3:**
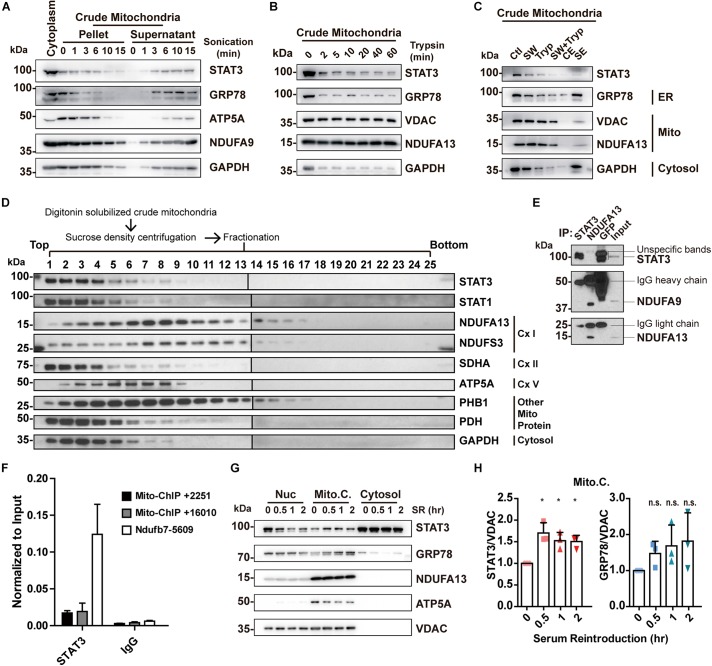
Re-examining the existence and function of ‘mitochondrial STAT3.’ **(A)** Purification of mitochondria by sonication. **(B)** Purification of mitochondria by trypsinization. **(C)** Purification of mitochondria by washing with high concentration of salt combined with trypsinization. GRP78 was used as the ER marker; ATP5A, NDUFA9, NDUFA13, and VDAC were used as the mitochondrial marker; GAPDH was used as the cytosolic marker. **(D)** Sucrose density centrifuge of digitonin-solubilized mitochondria followed by Western blot analysis of STAT3 and mitochondrial complexes protein. **(E)** Co-immunoprecipitation experiment in digitonin-solubilized crude mitochondria. **(F)** ChIP-qPCR detection of STAT3-binding on mitochondrial DNA in mouse embryonic stem cells. **(G)** Serum reintroduction experiment in Neuro2A cells. **(H)** Quantification of Western blot results **(G)** in three independent experiments. *p < 0.05, n.s. not significant; Ctl, control (washed with isotonic buffer); SW, salt-washed; Tryp, Trypsinized; CE, control elute; SE, salt-washed elute; Cx I, complex I; Cx II, complex II; Cx V, complex V; Nuc, nuclear fraction; Mito.C., crude mitochondrial fraction; SR, serum reintroduction.

Sonication methods resulted in the disruption of not only MAM but also mitochondria, as shown by a decreased level of both markers in the pellet (Figure 3A). In contrast, trypsinization had little deleterious effects on mitochondria integrity, but only achieved partial removal of MAM despite long incubations with enzyme of up to 60 min (Figure 3B). In separate studies, it has been reported that high salt washes of mitochondria followed by trypsinization can disrupt protein interactions and dissociate attached actin filaments (Boldogh et al., 1998). We attempted to examine if this method was able to remove the attached MAM from mitochondria. The results demonstrated that high salt washes combined with trypsinization was still unable to obtain pure mitochondria (Figure 3C). Though STAT3 remained in the mitochondria fractions obtained by all these methods, this may be explained by the presence of MAM fraction remnants, as indicated by the contamination of MAM and cytosol markers (Figures 3A–C).

In conclusion, the aforementioned methods fail to perfectly isolate pure mitochondria, and thus are unable to confirm the exclusive localization of STAT3 to mitochondria. At the same time, these results demonstrate that mitochondria-ER contacts may be resistant to sonication, trypsinization, and high salt washing.

### STAT3 Does Not Colocalize With Complex I, and Its Level Correlates With MAM Level

While we have demonstrated that STAT3 does not exist within mitochondria, other studies have shown that mitochondrial STAT3 binds to complex I or to mitochondrial DNA to modify mitochondrial metabolism (Wegrzyn et al., 2009; Macias et al., 2014). We attempted to separate mitochondrial protein complexes via sucrose density centrifugation. This result demonstrates that STAT3 does not exist within the complex I fraction (lanes 7, 8; Figure 3D). Meanwhile, we conducted co-immunoprecipitation assay to pull down endogenous STAT3 and a complex I subunit NDUFA13 from digitonin solubilized crude mitochondria. Pull down of STAT3 did not result in co-precipitation of complex I proteins (NDUFA9 and NDUFA13). Similarly, pull down of NDUFA13 achieved co-precipitation of NDUFA9, but not STAT3, suggesting the lack of interaction between STAT3 and complex I proteins (Figure 3E). In addition, our ChIP-qPCR experiments demonstrate that STAT3 does not bind to mitochondrial DNA in mouse embryonic stem cells (Figure 3F).

Previous studies have found that STAT3 levels in crude mitochondria increase after serum reintroduction following serum starvation (Xu et al., 2016). We hypothesized that this may result from increased MAM levels (Theurey et al., 2016). We performed serum reintroduction studies on Neuro2A cells. We found that reintroduction of serum increased the amount of STAT3 in the mitochondria fraction at as early as 30 min (Figures 3G,H), accompanied by an increased level of ER marker GRP78 in the same fraction (Figures 3G,H), suggesting that increased STAT3 levels may be due to the increase of MAM in the crude mitochondrial fraction.

### Other TFs or Signaling Proteins Are Present in MAM, but Not in Mitochondria

**FIGURE 4:**
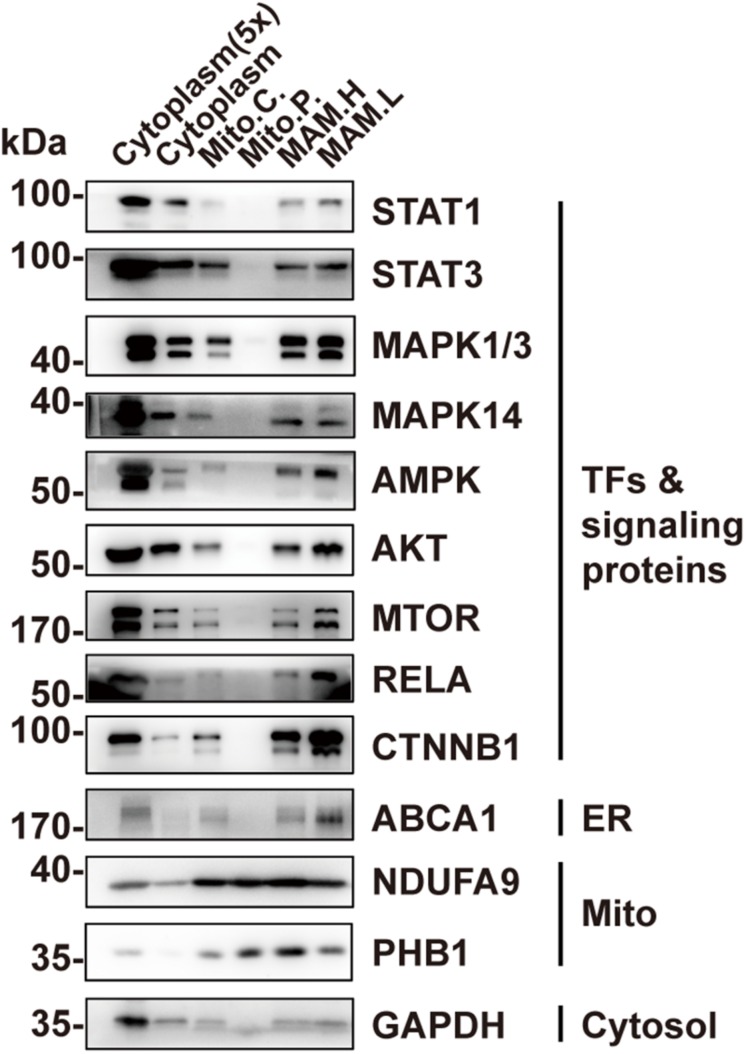
Other TFs or signaling proteins are present in MAM but not in mitochondria. Western blot analysis of the Percoll density centrifugation fractions from brain showed that transcription factors including STAT1, MAPKs, AMPK, AKT, mTOR, and RELA could only be found in the MAM fractions but not in pure mitochondria. Mito.C., crude mitochondrial fraction; Mito.P., pure mitochondrial fraction; MAM.H, heavy MAM; MAM.L, light MAM.

In addition to STAT3, a number of other TFs or signaling proteins have been previously found to localize in mitochondriawhere they are involved in regulating mitochondrial function, including STAT1 (Boengler et al., 2010; Bourke et al., 2013), mitogen-activated protein kinase 1/3 (MAPK1/3) (Poderoso et al., 2008; Galli et al., 2009), MAPK14 (Jang and Javadov, 2014; Yamauchi et al., 2014), AMPK (Zhao et al., 2019), AKT (Bijur and Jope, 2003; Ebner et al., 2017), mTOR (Ebner et al., 2017), as well as RELA (Cogswell et al., 2003; Johnson et al., 2011). We wondered whether, like STAT3, they actually localize in MAM instead of within mitochondria. We examined their localization in the pure mitochondrial fraction using Western blotting. The results demonstrate that none of these proteins were detectable in the pure mitochondria fraction (Figure 4). CTNNB1, which regulates mitochondrial metabolism but has never been found in mitochondria (Bernkopf and Behrens, 2018), was also found in the MAM fraction, suggesting a possible mechanism for MAM to regulate mitochondrial metabolism (Figures 4).

## Discussion

STAT3 has been shown to regulate mitochondrial metabolism by translocating to mitochondria (Wegrzyn et al., 2009; Macias et al., 2014). Initially, our study intended to investigate the functional mechanism of STAT3 in mitochondria. However, our work demonstrates that STAT3, along with some other nuclear TFs, are not present in the mitochondria, but instead are found in MAM. This suggests that STAT3 may regulate mitochondrial function via other mechanisms yet unknown.

Mitochondria-associated endoplasmic reticulum membrane plays an important role in mitochondrial metabolism, calcium signaling, lipid transport, and dynamics (Phillips and Voeltz, 2016; Theurey and Rieusset, 2017; Lee and Min, 2018; Rieusset, 2018). It has been reported that STAT3 can be found in the ER compartment, where it interacts with the IP3R and controls calcium efflux from the ER (Avalle et al., 2019). IR3R has also been found to form the MAM contact site by interacting with VDAC on the mitochondrial outer membrane (Vance, 2014). Thus, it is possible that MAM-STAT3 may regulate mitochondrial metabolism through modulating calcium transport. However, it is also possible that TFs and other nuclear proteins are only transported to the MAM fraction for degradation (Ma et al., 2017).

Several questions surrounding the “mitochondria-STAT3” theory have remained unanswered in the past decade. Firstly, what is the mechanism of the mitochondrial-import of STAT3 and other nuclear TFs? Protein import into mitochondria is restricted by the impermeable double membrane of mitochondria, and only proteins with mitochondrial signaling peptides can be imported into mitochondria through complex molecular machinery during translation (Harbauer et al., 2014; Wiedemann and Pfanner, 2017). However, little evidence exists that uncovers the mechanism of STAT3 import. It has been suggested that NDUFA13 is required for STAT3 import to mitochondria (Tammineni et al., 2013), yet the key mitochondrial channel responsible for this action has not been identified (Szczepanek et al., 2012). Until further evidence is uncovered, mitochondrial import of STAT3 is not well-supported by experimental results.

Secondly, how does “mitochondria-STAT3” regulate mitochondrial metabolism? Several mechanisms have been proposed, including its interaction with respiratory complex I/II, CypD and pyruvate dehydrogenase, as well as its regulation of the mitochondrial transcription (Wegrzyn et al., 2009; Macias et al., 2014; Carbognin et al., 2016; Xu et al., 2016; Meier et al., 2017). However, even if STAT3 was localized in mitochondria, it is unlikely to influence mitochondrial metabolism through direct interaction with mitochondrial proteins or genome, considering the stoichiometric difference between STAT3 (∼10^2^ molecules/cell) and mitochondrial protein (6 × 10^6^ molecules of complex I or II/cell) or mitochondrial genome (1,000∼5,000 copies/cell) (Bogenhagen and Clayton, 1974; Shmookler Reis and Goldstein, 1983; Phillips et al., 2010). Proposed mechanisms of STAT3 activity by other groups are largely based on the overexpression strategies of “mito-STAT3” (STAT3 fused with a mitochondrial signaling peptide at the N-terminus) which employs the assumption that STAT3 localizes to mitochondria (Wegrzyn et al., 2009; Xu et al., 2016; Meier et al., 2017). These results may prove to be artificial or artifactual if this assumption proves to be false.

In summary, our results suggest that STAT3, along with other TFs or signaling proteins with previously described mitochondrial localization, actually localize in MAM, contrary to prior reports. Extra attention must be paid to the localization of these factors in the future due to the inherent subjectivity of cell fractionation.

## Materials and Methods

### Animal

Wild type C57BL/6j mice were euthanized by CO_2_ overdose before dissection and tissues or embryos collection. All animals used in the study were maintained in the Specific-pathogen-free animal facility. All procedures were performed under the approval of the Sun Yat-sen University Institutional Animal Care and Use Committee.

### Cell Culture

Primary neural progenitors were isolated from embryonic Day (E) 14 mouse embryos and cultured in serum-free medium [Dulbecco’s Modified Eagle Medium/Nutrient Mixture F-12 (DMEM/F12)] with B27 supplement and epidermal growth factor (EGF)/fibroblast growth factor (FGF). Astrocytes were isolated from E15 embryos and were cultured in DMEM with 10% FBS. Mouse embryonic stem cells were maintained in DMEM supplemented with 15% FBS, GlutaMax, NEAA, beta-mercaptoethanol and LIF. Neuro2A cells were cultured and passaged in DMEM with 10% FBS. Serum reintroduction experiments were performed on Neuro2A cells by subjecting cells to serum-free culture for 6 h, followed by serum reintroduction at various time points prior to collection.

### Cell Fractionation by Sucrose Density Centrifugation

Cells cultured in a 10-cm dish were scraped down in 0.5 ml isotonic buffer (5 mM HEPES, 250 mM sucrose, 0.1 mM EDTA) and transferred to an Eppendorf tube. The dishes were then washed with 0.5 ml isotonic buffer, which was then pooled together. Cells were homogenized by repeated passes through a 25-gauge needle attached to a 1-ml syringe 15 times. 100 μl of the homogenate was saved as the whole cell lysate control. The remaining homogenate was centrifuged at 750 × *g* for 5 min to remove intact cells and nucleus. The cytoplasm was collected from the supernatant, and 100 μl was saved as control. The cytoplasmic fraction was then layered on a sucrose gradient (7 ml 1.0 M sucrose, 3 ml 1.5 M sucrose, each contained 5 mM HEPES and 0.1 mM EDTA) in a 14 ml ultracentrifugation tube. The tubes were then subjected to ultracentrifugation in a SW40Ti rotor at 28,000 rpm for 4 h. The resulting gradient was fractionated and subjected to Western blot analysis.

### Isolation of the Pure Mitochondria and MAM Fractions by Percoll Density Centrifugation

The isolation of the pure mitochondria and MAM fractions from mouse tissue was performed as previously described with slight modification (Wieckowski et al., 2009; Schreiner and Ankarcrona, 2017). The same procedure was applied to all three tissues (brain, liver, muscle). Tissues from one mouse were used in a single experiment. Briefly, tissues were dissected from euthanized mice, followed by homogenization. Homogenates were then centrifuged at 750 × *g* for 5 min to remove unbroken cells and nucleus. An aliquot of the cytoplasmic fraction collected from the supernatant was then centrifuged at 10,000 × *g* for 5 min to obtain the crude mitochondria fraction (Mito.C).

The rest of the cytoplasmic fraction was layered on 10 ml 30% Percoll buffer in a 14-ml ultracentrifugation tube and centrifuged at 96,000 × *g* for 30 min using SW-40Ti rotor (Beckman). The resulting gradient was confirmed to contain two yellow/white bands. The top band (MAM fraction) and the bottom band (pure mitochondria) were separately collected. The Cytosol fraction was collected from the clear layer above the top band. In the liver fractionation experiment, an extra cytosol layer rich in fat was also collected (Cytosol.F). The cytosol fractions collected in 1.5-ml tube were then centrifuged at 10,000 × *g* for 10 min to remove potential MAM contamination.

To remove excess Percoll and further purify the fractions, both mitochondrial and MAM fractions were diluted with 12 ml mitochondria resuspension buffer (MRB) in 14-ml ultracentrifugation tubes. The mitochondrial fraction was centrifuged at 6,000 × *g* for 15 min, the pellet was collected as pure mitochondria (Mito.P). Subsequently the supernatant was centrifuged at 100,000 × *g* for 60 min, the pellet was then collected as ‘light’ pure mitochondria (Mito.P.L); this fraction might represent smaller, lighter mitochondria, and could be contaminated with MAM. Noted that only from the liver tissue could we obtain the visible Mito.P.L fraction. Meanwhile, the MAM fraction was centrifuged at 6,000 × *g* for 15 min, the pellet was collected as the ‘heavy’ MAM (MAM.H), which might contain less ER and is therefore heavier. The supernatant was centrifuged at 100,000 × *g* for 60 min and the pellet was collected as the MAM fraction. All the pellets (Mito.P/MAM fractions) were collected in 1 ml MRB and transfer to 1.5-ml tube, followed by centrifugation at 10,000 × *g* for 10 min. The supernatants were removed and the pellets were resuspended again with MRB. However, after centrifugation of the liver MAM fraction, the supernatant remained cloudy. This fraction was also collected as ‘light’ MAM fraction (MAM.L).

The protein concentration from each samples was measured by the Bradford assay. For Western blot analyses, the same amount of protein was loaded for all samples except the cytoplasmic fraction: it was loaded at five times the amount of the other fractions.

### Purification of Mitochondria by Sonication, Trypsinization and Washing With High Concentration of Salt

The crude mitochondria were obtained from mouse brain as described above. Mitochondria from one mouse brain was used in each experiment.

To purify mitochondria by sonication, the crude mitochondria were subjected to sonication by the Scientz08-II sonicator at 50% power for 0 min, 1 min, 3 min, 6 min, 10 min, and 15 min, respectively. The resulting suspensions were centrifuged at 10,000 × *g* for 5 min. The pellet and supernatant were collected separately. The pellets were washed once and resuspended in MRB.

To purify mitochondria by trypsinization, 2 μg/ml trypsin was added to crude mitochondrial fractions. The trypsinization was stopped at 2 min, 5 min, 10 min, 20 min, 40 min, and 60 min, respectively by FBS addition (same volume). The resulting suspensions were centrifuged at 10,000 × *g* for 5 min and the pellets were collected and washed once and resuspended in MRB.

To purify mitochondria by washing with high concentration of salt, the crude mitochondrial fraction was incubated in MRB supplemented with 1 M KCl and 2 mM MgCl_2_ for 15 min on ice, and centrifuged at 10,000 × *g* for 5 min. The salt-washed mitochondria were recovered in the pellet, while the eluted protein was recovered in the supernatant. Subsequently, salt-washed mitochondria were incubated in 2% trypsin for 30 min. Mitochondria were then collected by centrifugation, washed once and resuspended in MRB.

### Mitochondrial Complexes Sucrose Density Centrifugation

The mitochondrial fractionation protocol was adopted from Shinzawa-Itoh et al. (2016). Mitochondria from one mouse brain was used in one experiment. The crude mitochondria were isolated and incubated with 2% digitonin (detergent:protein = 4:1) on ice for 30 min. The sample was then centrifuged at 65,000 × *g* for 10 min to remove the insoluble fractions. The supernatant was then loaded onto a sucrose gradient (1.3 M, 1.4 M, 1.5 M, 1.55 M, 1.6 M), and then centrifuged at 105,000 × *g* for 24 h. The resulting gradient was then fractionated from top to bottom.

### Co-immunoprecipitation

Crude mitochondria were solubilized by incubation of 2% digitonin (detergent:protein = 4:1) on ice for 30 min, which was then diluted five times with MRB. The Protein A/G beads (SCBT) were incubated on ice for 30 min with STAT3 antibody (SCBT), NDUFA13 antibody (Invitrogen) and GFP antibody (SCBT), respectively, then washed once with 1 ml MRB. The solubilized mitochondria were then added to the antibody-coated beads and incubated overnight at 4°C. The beads were then collected by centrifugation at 100 × *g* for 1 min, washed for four times with MRB, then boiled in SDS sample buffer for Western blot analysis.

### ChIP-qPCR

Mouse embryonic stem cells were crosslinked with formaldehyde at a final concentration of 1% for 10 min followed by quenching with glycine. Chromatin extracts were fragmented by sonication and pre-cleared with protein G Dynabeads, then subsequently precipitated with anti-STAT3 antibody (Santa Cruz), anti-p65 antibody (Santa Cruz), or normal rabbit IgG (Santa Cruz) overnight at 4°C. After washing and elution, crosslink reversal was done by incubating at 65°C for 8 h. The eluted DNA was purified and analyzed by qPCR with primers specific to the predicted STAT3 binding site. qPCR experiment was carried out using SYBR Green qPCR kit from KAPA and Applied Biosystems 7500 Real PCR System. Samples were assayed in duplicate. Primers sequences are: Mt-ChIP-2F, tggggtgacctcggagaat; Mt-ChIP-2R, cctagggtaacttggtccgt; Mt-ChIP-13F, ccgcaaaacccaatcacctaag; Mt-ChIP-13R, ttggggtttggcattaagagga; mNdufb7-F, tctgttaaatgtcacccgtcct; mNdufb7-R, acttttacacctggtacccaaca.

### Cell Fractionation by Differential Centrifugation

After serum reintroduction experiment, cells cultured in a 10-cm dish were scraped down in 0.5 ml isotonic buffer (5 mM HEPES, 250 mM sucrose, 0.1 mM EDTA) and transferred to an Eppendorf tube. The dishes were then washed with 0.5 ml isotonic buffer, which was then pooled together. Cells were homogenized by repeated passes through a 25-gauge needle attached to a 1-ml syringe 15 times. The remaining homogenate was centrifuged at 750 × *g* for 5 min. The pellet (P1) contained intact cells and nucleus. The cytoplasm collected from the supernatant was then centrifuged at 10,000 × *g* for 5 min, and pellet [the crude mitochondria fraction (Mito.C)] was washed once with the isotonic buffer. The supernatant was collected as the cytosolic fraction. To separate nucleus from the intact cells, P1 was resuspended in isotonic buffer containing 0.1% NP-40, triturated and centrifuged at 750 × *g* for 5 min. The resulting pellet (the nucleus fraction) was washed in isotonic buffer once.

### Western Blotting

Protein samples were lysed in 5 × SDS sample buffer containing beta-mercaptoethanol, boiled at 95°C for 10 min and loaded into each well for SDS-PAGE. Samples were then transferred to PVDF membrane (Thermo) and immunoblotted using anti-NDUFA9 (Invitrogen), anti-NDUFS3 (Invitrogen), anti-NDUFA13 (Invitrogen), anti-ATP5A (Invitrogen), anti-SDHA (CST), anti-VDAC (CST), anti-HSP60 (CST), anti-PHB1 (CST), anti-PDH (CST), anti-GAPDH (Sigma), anti-GRP78 (SCBT), anti-ABCA1 (SCBT), anti-FLOT1 (CST), anti-STAT3 (CST), anti-STAT1 (SCBT), anti-AMPK (CST), anti-ERK1/2 (CST), anti-p38 (CST), anti-SYP (Abcam), anti-ACSL4 (Abcam), all diluted in 5% BSA:TBST at 1:1000, followed by appropriate HRP-conjugated secondary antibodies (Thermo) incubation (diluted in 5% non-fat milk:TBST at 1:10,000), and developed using the SuperSignal^TM^ West Femto Maximum Sensitivity Substrate (Thermo).

### Immunofluorescence

Astrocytes isolated from E15 and cultured on poly-D-lysine-coated coverslips were stained with 5 μM MitoTracker DeepRed to label mitochondria. Alternatively, cells were transfected with ER-dsRed expression vector 1 day prior to fixation to label ER. Cells were fixed with 4% paraformaldehyde for 15 min and were then permeabilized by ice-cold methanol for 5 min, followed by PBS rinse for 10 min. The cells were then blocked with 5% bovine serum albumin in PBS with 0.3% TritonX-100 for 1 h, followed by primary antibody [rabbit-anti-HSP60 (CST), rabbit-anti-LC3B (CST), rabbit-anti-LAMP1 (CST), rabbit-anti-ACSL4 (Abcam), mouse-anti-STAT3 (CST), both diluted at 1:200 in the blocking buffer] incubation at 4°C overnight. Then, the cells were incubated in appropriate secondary antibodies [donkey-anti-rabbit-AF488, donkey-anti-rabbit-647, donkey-anti-mouse-488, donkey-anti-mouse-AF568 or donkey-anti-mouse-AF647 (Thermo), all diluted at 1:200 in the blocking buffer] for 1 h at room temperature. After washing three times in TBST, the cells on the coverslip was mounted using Prolong Gold with DAPI (Thermo) and subjected to confocal microscopy observation using the Zeiss LSM880 with Airyscan system. Images were captured using a 63x/1.4NA oil immersion objective. The colocalization analysis was performed with Fiji software using the Coloc 2 plugin.

### Statistic

Statistical significance was determined by Student’s *t*-test using GraphPad Prism 6.01. The *p*-value < 0.05 was considered significant. Unless otherwise specified, data were presented as mean and the standard deviation (mean ± SD).

## Data Availability Statement

The raw data supporting the conclusions of this manuscript will be made available by the authors, without undue reservation, to any qualified researcher.

## Ethics Statement

The animal study was reviewed and approved by the Sun Yat-sen University Institutional Animal Care and Use Committee.

## Author Contributions

YS contributed to the methodology, visualization, investigation and writing the original draft. XH contributed to conceptualization and investigation. ZH contributed to investigation. TH contributed to validation. YX contributed to supervision. CY contributed to supervision, review and editing the manuscript.

## Conflict of Interest

The authors declare that the research was conducted in the absence of any commercial or financial relationships that could be construed as a potential conflict of interest.

## Abbreviations

ABCA1: ATP-binding cassette transporter
ACSL4: long chain fatty acyl-CoA synthetase 4
AKT: protein kinase B
AMPK: AMP-activated protein kinase
CTNNB1: catenin beta 1
CypD: cyclophilin D
ER: endoplasmic reticulum
GAPDH: glyceraldehyde 3-phosphate dehydrogenase
GRP78: glucose-regulated protein 78
HSP60: heat shock protein 60
IP3R: inositol trisphosphate receptor
MAM: mitochondria-associated endoplasmic reticulum membrane
MAPK: mitogen-activated protein kinase
mTOR: mammalian target of rapamycin
NDUFA13: NADH:ubiquinone oxidoreductase subunit A13
RELA: NF- κ B p65 subunit
STAT3: signal transducer and activator of transcription 3
TF: transcription factor
VDACs: voltage-dependent anion channels.
